# Comparing cellular performance of *Yarrowia lipolytica* during growth on glucose and glycerol in submerged cultivations

**DOI:** 10.1186/2191-0855-3-58

**Published:** 2013-10-03

**Authors:** Mhairi Workman, Philippe Holt, Jette Thykaer

**Affiliations:** 1 Department of Systems Biology, Building 223, Soeltofts Plads, Technical University of Denmark, 2800 Kgs. Lyngby, Denmark

**Keywords:** *Y. lipolytica*, Glycerol, Submerged cultivation, Oxygen limitation, Polyols

## Abstract

*Yarrowia lipolytica* is an attractive host for sustainable bioprocesses due to its ability to utilize a variety of carbon substrates and convert them to a range of different product types (including lipids, organic acids and polyols) under specific conditions. Despite an increasing number of applications for this yeast, relatively few studies have focused on uptake and metabolism of carbon sources, and the metabolic basis for carbon flow to the different products. The focus of this work was quantification of the cellular performance of *Y. lipolytica* during growth on glycerol, glucose or a mixture of the two. Carbon substrate uptake rate, growth rate, oxygen utilisation (requirement and uptake rate) and polyol yields were estimated in batch cultivations at 1 litre scale. When glucose was used as the sole carbon and energy source, the growth rate was 0.24 h^-1^ and biomass and CO_2_ were the only products. Growth on glycerol proceeded at approximately 0.30 h^-1^, and the substrate uptake rate was 0.02 mol L^-1^ h^-1^ regardless of the starting glycerol concentration (10, 20 or 45 g L^-1^). Utilisation of glycerol was accompanied by higher oxygen uptake rates compared to glucose growth, indicating import of glycerol occurred initially via phosphorylation of glycerol into glycerol-3-phosphate. Based on these results it could be speculated that once oxygen limitation was reached, additional production of NADH created imbalance in the cofactor pools and the polyol formation observed could be a result of cofactor recycling to restore the balance in metabolism.

## Introduction

With increasing focus on the development of sustainable technologies, the necessity for converting alternative, cheaper and waste carbon sources is emerging. Due to increased production of biodiesel, glycerol has become available in quantities relevant for large scale bioprocesses (Thompson and He 2006Johnson and Taconi 2007da Silva et al. 2009), while glucose, the most abundant carbon source in nature, is typically applied in industrial scale bioprocesses. The challenge is to find efficient microbial hosts for the conversion of these substrates to value added products, meeting the demands of modern society for fuel and energy, biochemicals and pharmaceuticals.

The traditionally applied yeast cell factory, *Saccharomyces cerevisiae*, exhibits a clear preference for glucose as a carbon source (μmax ≈ 0.4 h^-1^), and is highly adapted to its utilisation (Walker 1997). With the evolution of high affinity glucose transporters succeeded by the whole genome duplication and the evolution of ethanol tolerance, *S. cerevisiae* gained a significant advantage (Kellis et al. 2004Conant and Wolfe 2007). When cultivating this yeast on glucose, the strain exhibits an overflow metabolism caused by excessive amounts of glucose entering glycolysis, resulting in secretion of high amounts of ethanol, compared to other yeasts. The advantage of ethanol tolerance and the high affinity for glucose has been exploited by industry for decades, and has also led to a high level of understanding of the strain and its metabolism (Nevoigt 2008). However, this high level of adaptation has resulted in a strain that is not naturally efficient in its conversion of alternative carbon sources such as glycerol (μmax = 0.02 h^-1^, for CEN.PK 113-5D, Liu et al. 2013; 0.008 h^-1^,Merico et al. 2010) and xylose, the second most abundant sugar in nature, cannot be utilised for growth by wild type strains (Hahn-Hägerdal et al. 2007van Maris et al. 2007). Therefore *S. cerevisiae* is not such an attractive cell factory when alternative carbon sources are being considered.

The non-conventional yeast *Yarrowia lipolytica* has a high capability for growing on a range of carbon sources from glucose, fructose and xylose to glycerol and hydrophobic substrates (Coelho et al. 2010Fickers et al. 2005;da Silva et al. 2012). These substrates can be converted into value added products including citric acid (da Silva et al. 2012Papanikolaou et al. 2002Levinson et al. 2007Rymowicz et al. 2010), sugar alcohols (Rymowicz et al. 2009Tomaszewska et al. 2012) and single cell oils (Beopoulos et al. 2009Fontanille et al. 2012), demonstrating the versatility of the organism to produce small organic compounds of industrial relevance. Derivation of energy from different types of carbon substrates infers entry into the central metabolism at different points, and in *Y. lipolytica* suggests a natural adaptability in the mechanisms for generation of energy. From a biotechnological point of view, it is interesting to understand these mechanisms and capabilities to further exploit them for industrial scale processes.

Although *Y. lipolytica* shows an interesting extracellular product spectrum, data on physiological conditions required to support production of the various products and the underlying mechanisms from a metabolic point of view is limited. The physical and chemical conditions leading to production of the different products types is difficult to define precisely from the literature as a range of cultivation modes have been employed and often the addition of complex medium components complicate accurate interpretation of cause and effect. In general, pH, oxygen saturation in the medium and the ratio of carbon to nitrogen appear to be the major factors shown to influence citric acid production and lipid accumulation.

The focus of this study was to assess the physiology of *Y. lipolytica* when grown on glucose and glycerol as sole carbon sources or on a mixture of the two. *Y. lipolytica* has been well studied for the production of a range of different types of metabolites. The interest here was polyol formation. Our aim was to make a quantitative study of the physiology of this yeast under different growth conditions in order to understand and begin to map the metabolism from substrate to product of interest. An understanding of these processes can form the basis for future metabolic engineering of this yeast and further its development as an industrial workhorse.

## Materials and methods

### Micro-organism

*Yarrowia lipolytica* IBT 446, was used through-out this study. The strain was obtained from the culture collection at the Department of Systems Biology, Technical University of Denmark as an isolate from feta cheese. The strain cryostock was maintained at −80°C in 50% (w/w) of glycerol.

### Liquid medium

*Y. lipolytica* IBT 446 was cultivated in a defined minimal medium with the following composition: (NH_4_)_2_SO_4_, 5.0 g L^-1^; KH_2_PO_4_, 3.0 g L^-1^, MgSO_4_.7H_2_O, 0.5 g L^-1^; Antifoam 298 (Sigma-Aldrich), 0.05 mL L^-1^; trace metal solution, 1 mL L^-1^ (composed of: FeSO_4_.7H_2_O, 3 g L^-1^; ZnSO_4_.7H_2_O, 4.5 g L^-1^; CaCl_2_.6H_2_O, 4.5 g L^-1^; MnCl_2_.2H_2_O, 0.84 g L^-1^; CoCl_2_.6H_2_O, 0.3 g L^-1^; CuSO_4_.5H_2_O, 0.3 g L^-1^; Na_2_MoO_4_.2H_2_O, 0.4 g L^-1^; H_3_BO_3_, 1 g L^-1^; KI, 0.1 g L^-1^; Na_2_EDTA.2H_2_O, 15 g L^-1^); 1 mL L^-1^ vitamin solution (composed of: d-biotin, 25 mg L^-1^; Ca-pantothenate, 0.5 g L^-1^; thiamin-HCl, 0.5 g L^-1^; pyridoxin-HCl, 0.5 g L^-1^; nicotinic acid, 0.5 g L^-1^; p-aminobenzoic acid, 0.1 g L^-1^; m-inositol, 12.5 g L^-1^). All chemicals used were of analytical grade. pH of the medium was adjusted to 6.5 by 2 M NaOH prior to autoclavation.

Four sets of cultivations were performed with the following carbon source(s) and concentration(s): glucose, 20 g L^-1^ (0.65 cmol L^-1^), glycerol, 20 g L^-1^ (0.65 cmol L^-1^), glycerol 45 g L^-1^ (1.5 cmol L^-1^); glycerol and glucose, 10 g L^-1^ each (0.32 cmol L^-1^ each). The term cmol L^-1^ is used for ease of comparison between glucose and glycerol.

### Preparation of inoculum

The inoculum for batch cultivations was prepared as a pre-culture in minimal medium, containing the same primary carbon source as the intended batch cultivation. Baffled 250 mL shake flasks containing 50 mL minimal medium were inoculated with a *Y. lipolytica* colony from an agar plate. The shake flasks were incubated at 30°C and 160 rpm in a rotary shaker (Thermo Fisher Scientific, United States) and the pre-culture was harvested in mid exponential phase and used for inoculation of batch cultivations to an initial OD600 of 0.2.

### Batch cultivations

All cultivations were performed in 1 L fully instrumented and automatically controlled BIOSTAT® Q plus fermenters (Sartorius Stedim Biotech S.A, Germany) using a working volume of 1 L. Temperature and stirring rate were monitored and controlled at 30°C +/− 1°C, 600 rpm, respectively whereas pH was controlled at and 4.5 +/− 0.1 by automatic addition of 2 M NaOH and 1 M HCl. The bioreactor was sparged with atmospheric air maintaining aeration of 1 volume per volume per minute (vvm) (1 standard liter per minute (slpm)). Partial oxygen pressure (pO_2_) was constantly monitored online. The exhaust gas was measured in a gas analyzer (Prima PRO Process Mass Spectrometer, Thermo Fisher Scientific) quantifying the concentrations of oxygen and carbon dioxide.

### Cell mass concentration

The cell mass concentration was determined both through measurements of dry weight and spectrophotometrically. Optical density of samples from the cultivations was measured at 600 nm on a Shimadzu UV-mini 1240 spectrophotometer. Dry weight was measured using 0.45 μm nitrocellulose filters (Sartorius Stedium, Germany). The filters were dried in a microwave oven at 150 W for 20 min before and after filtration and the weights of the filters were noted without and with biomass after cooling down in a desiccator.

### Analytical methods

HPLC was performed to quantify extracellular concentrations of the substrates (glucose and glycerol) and polyols (erythritol, mannitol and arabitol). 1 ml culture sample was filtrated through a Q-Max® Ca-Plus Syringe Filter with a pore size of 0.45 μm to a HPLC vial which was stored at 4°C. The concentrations of glucose, glycerol, citric acid, mannitol, erythritol and arabitol were obtained with a Bio-Rad Aminex HPX-87H column coupled to a RI detector. The solvent used was 5 mM H_2_SO_4_ with a flow velocity of 0.6 ml/min at 60°C.

### Data analysis

All experiments were performed in duplicate. The yield coefficients were estimated through linear regression of product as a function of substrate consumption with a regression correlation of above 0.95. Yxo values were similarly determined through linear regression of consumed oxygen as a function of biomass concentration. Maximum specific growth rate (μ max) was estimated from an exponential fit of biomass concentration as a function of time during exponential growth. The specific uptake rates of oxygen r_o_ were estimated from Yxo by multiplication with μmax. Standard errors on the mean of at least two duplicate experiments were calculated and have been included in the assessment of data. For conversion of gram dry weight to c-mole basis, a molecular weight of 23.57 g DW cmol ^-1^ was used.

## Results

### Cultivation profiles

**Figure 1:**
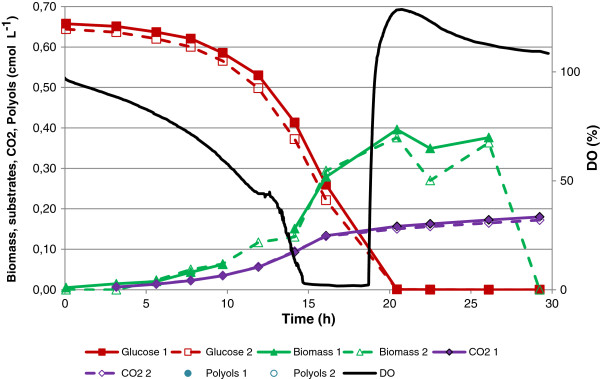
**Batch cultivation of*Y. lipolytica*with 20 g L**^**-1**^**glucose (0.65 cmol L**^**-1**^**) showing substrate, biomass and product concentration as a function of time.**

**Figure 2:**
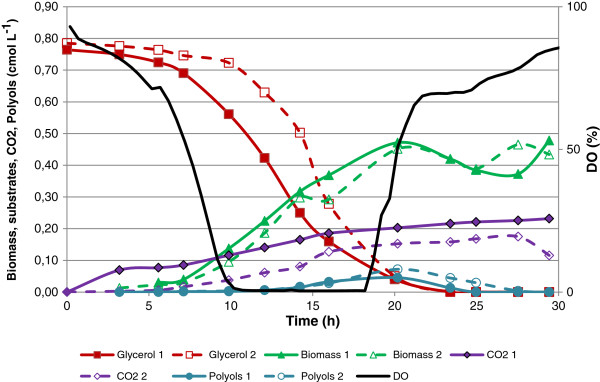
**Batch cultivation of*Y. lipolytica*with 20 g L**^**-1**^**glycerol (0.65 cmol L**^**-1**^**) showing substrate, biomass and product concentration as a function of time.**

An initial set of cultivations was performed using glucose (Figure 1) and glycerol (Figure 2) as the sole carbon and energy source, at a concentration of 20 g L^-1^ (0.65 cmol L^-1^). During the batch culture with glucose as carbon source, the oxygen became limiting at around 15 hours, and the only products of the process were biomass and CO_2_ as no metabolite production was observed. The time profile for the cultivation is shown in Figure 1. A similar process with glycerol as the sole carbon and energy source, at 20 g L^-1^ (0.65 cmol L^-1^) was then performed, to compare the cellular performance. Using glycerol as carbon source, the batch cultivation became oxygen limited after 10 hours, and halfway into the phase with oxygen limitation low amounts of polyols, in the form of mannitol and arabitol, were produced (Figure 2).

**Figure 3:**
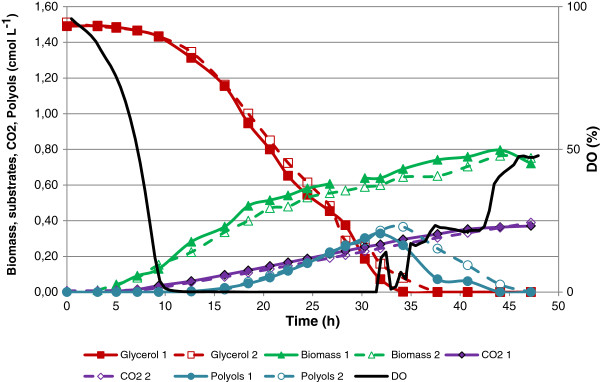
**Batch cultivation of*Y. lipolytica*with 40 g L**^**-1**^**glycerol (1.50 cmol L**^**-1**^**) showing substrate, biomass and product concentration as a function of time.**

To challenge the glycerol metabolism during oxygen limited conditions, a third single substrate cultivation was performed with 45 g L^-1^ glycerol (1.50 cmol L^-1^). A plot of the cultivation data can be seen in Figure 3. All three single substrate cultivations were carried out in duplicates and the reproducibility with respect to growth, glycerol uptake and metabolite production can be seen in Figures 1, 2, and 3. For the two cultivations using glycerol as carbon source, three distinct phases can be described (Figures 2 and 3). Phase 1 was characterised by exponential growth with carbon and oxygen being available in excess. Phase 2 was characterised by oxygen limitation and metabolite formation, with production of the polyols, primarily mannitol and arabitol, being observed. A third phase followed during which metabolites were consumed. In both cultures, the exponential growth phase finished after approximately 12 hours but in the culture with the higher glycerol concentration, both phase 2 and 3 were prolonged compared with the 20 g L^-1^ glycerol culture. In the culture with glucose as carbon source (Figure 1), three phases were also observed but unlike the glycerol cultures, no polyol or metabolite production was measured in the oxygen-limited phase, which was also shorter (4 h) compared to the glycerol processes (8 h). In addition, the exponential growth phase was prolonged due to the lower maximum specific growth rate of *Y. lipolytica* on glucose (Table 1).

**Table 1:** Quantitative data for the cultivations on glucose, glycerol and a mixture of glucose and glycerol.

	**Glucose**	**Glycerol**	**Glycerol**	**Glucose + Glycerol**
	**0.65 cmol L**^**-1**^	**1.5 cmol L**^**-1**^	**0.65 cmol L**^**-1**^	**0.65 cmol L**^**-1**^
**Growth rate**				
μmax (h^-1^)	0.24 ± 0.01	0.32 ± 0.01	0.30 ± 0.01	0.38 ± 0.01
**Oxygen consumption**				
Yxo (mol cmol^-1^)	0.42 ± 0.04	0.44 ± 0.03	0.43 ± 0.01	0.33 ± 0.01
r_o_ (mol gDW^-1^ h^-1^)	0.11 ± 0.01	0.14 ± 0.01	0.14 ± 0.01	0.13 ± 0.01
**Glycerol uptake**				
q_glycerol_(mol L^-1^ h^-1^)	N/A	0.020 ± 0.001	0.019 ± 0.001	0.014 ± 0.002
**Yield coefficients**				
Ysx (cmol cmol^-1^)	0.69 ± 0.03	0.64 ± 0.00	0.61 ± 0.01	0.76 ± 0.04
Ysc (cmol cmol^-1^)	0.30 ± 0.01	0.17 ± 0.01	0.23 ± 0.00	0.18 ± 0.01
Ysm (cmol cmol^-1^)	N/A	0.24 ± 0.00	0.17 ± 0.00	0.06 ± 0.01
**Total**	**0.99 ± 0.03**	**1.05 ± 0.01**	**1.01 ± 0.01**	**1.00 ± 0.04**

N/A: Not applicable.

**Figure 4:**
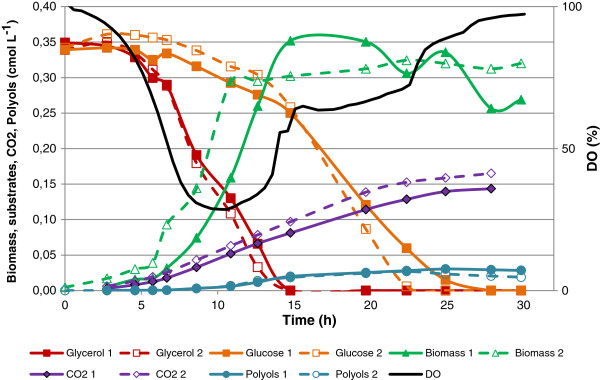
**Batch cultivation of*Y. lipolytica*with 10 g L**^**-1**^**glucose (0.37 cmol L**^**-1**^**) and 10 g L**^**-1**^**glycerol (0.37 cmol L**^**-1**^**) corresponding to a total carbon concentration of 0.65 cmol L**^**-1**^**showing substrate, biomass and product concentration as a function of time.**

Figure 4 shows the cultivation time profile for double substrate experiment where *Y. lipolytica,* was cultivated on equal amounts of glycerol and glucose, 10 g L^-1^, corresponding to a total carbon concentration of 0.65 cmol L^-1^ (and thus comparable to the single carbon substrate processes at 0.65 cmol L^-1^; Figures 1 and 2). The yeast favoured glycerol and this carbon source was metabolised and depleted before glucose. However, from the substrate consumption profile (Figure 4) it was observed that in the time frame between 7 and 13 hours, co-consumption of the two carbon sources occurred. A clear distinction was noticed between the phase of growth on mainly glycerol and that on glucose alone, as the oxygen saturation rose abruptly after depletion of glycerol. After depletion of most of the glycerol, polyol formation occurred, which continued after glycerol was fully depleted. The cultures did at no point reach oxygen limitation when both substrates were available.

From the cultivation process data, the maximum specific growth rate was estimated for each single substrate experiment and for the double substrate culture. As shown in Table 1, *Y. lipolytica* was capable of growing on glycerol with a maximum specific growth rate (μmax) of 0.31 h^-1^, whereas μmax during growth on glucose was estimated to 0.24 h^-1^, representing a 23% reduction.

### Oxygen utilisation and glycerol uptake

Oxygen availability was shown to be a key parameter in the glycerol cultivations, and for the production of polyols. The cultures reached oxygen limitation after approximately 12 hours for the glycerol processes (Figures 2 and 3), independent of glycerol concentration, indicating supply of oxygen determined the rate at which glycerol could be utilised for growth. By increasing the oxygen availability (in cultures where dissolved oxygen was maintained at 25, 50 and 100% respectively), it was shown that the exponential growth phase could be prolonged (to 16 hours), see Additional file 1. The yield coefficients Yxo (moles of O_2_ consumed per cmol of biomass produced) were estimated during growth on the different carbon sources, Table 1. Interestingly, Yxo was the same for all the single substrate cultures, whereas a 25% reduction in Yxo was estimated for the combined substrate cultivation. The specific uptake rates of oxygen r_o_ were estimated from Yxo by multiplication with μmax. As indicated in Table 1, r_o_ ranged between 0.11-0.14 moles O_2_ cmol^-1^ h^-1^ for all the experiments where the specific oxygen uptake rate was lower in the glucose cultivations.

The volumetric glycerol uptake rates in the glycerol cultivations were estimated by linear regression of the substrate concentration as a function of time, and results are summarised in Table 1. The volumetric glycerol uptake rates are given in moles L^-1^ h^-1^ (rather than Cmoles) to account for the uptake of molecules of the carbon sources used. The uptake rates were similar for the 0.65 cmol L^-1^ and 1.5 cmol L^-1^ glycerol cultivations at 0.019 moles L^-1^ h^-1^ ± 0.001 and 0.021 moles L^-1^ h^-1^ ± 0.001 respectively. In comparison to the cultivations with glycerol as the sole carbon source, the volumetric substrate uptake rate of glycerol in the combined glycerol and glucose cultivation was reduced to 0.014 ± 0.002 moles L^-1^ h^-1^. To test whether this was an effect of a lower glycerol concentration, or an alternative effect based on oxygen availability or the presence of glucose, an additional bioprocess was performed at 0.325 cmol L^-1^ glycerol (duplicate experiments with main findings illustrated in Additional file 2). The glycerol uptake rate was estimated to be 0.019 moles L^-1^ h^-1^ ± 0.001, and thus the reduced uptake rate was not likely to be due to the reduced concentration of glycerol.

### Yield coefficients

An overview of the yield coefficients of biomass (Ysx), carbon dioxide (Ysc) and polyols (Ysp) on substrate is presented in Table 1. As shown in the last row of the table, the carbon balance closes for all four sets of cultivations. In the glucose culture 69% of the carbon was used for biomass formation whereas the residual 30% was converted into carbon dioxide. For the cultures run with glycerol as carbon source, similar biomass yields (Ysx) were obtained. The CO_2_ yield (Ysc) was lower for the high glycerol culture whereas the polyol yield was higher compared to the low glycerol process. The culture with the combined carbon sources had both the highest growth rate and the highest biomass yield (Ysx). The carbon dioxide yield (Ysc) was similar to the high glycerol culture but the polyol yield (Ysp) was significantly lower than observed for the single substrate cultures on glycerol. Thus, both the oxygen limitation and the nature and amount of carbon source had an influence on the growth rate of *Y. lipotylica* as well as the carbon distribution.

**Figure 5:**
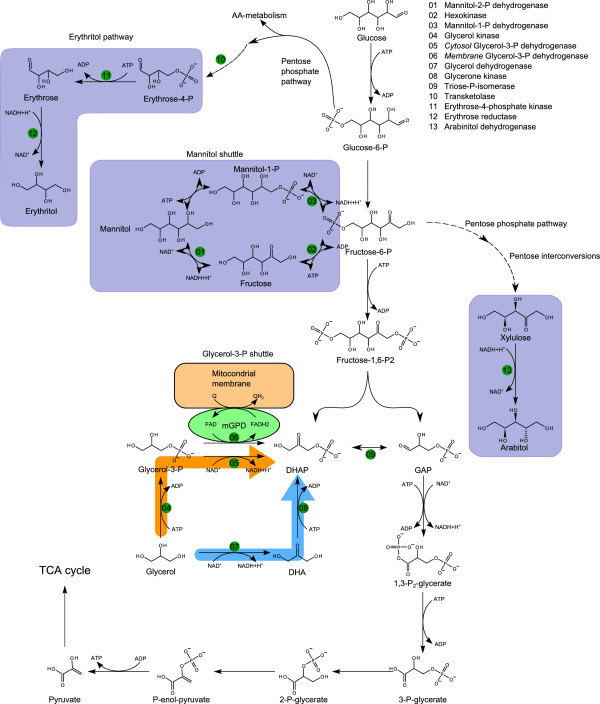
**Pathways of glucose and glycerol utilisation in*Yarrowia lipolytica*towards the mannitol shuttle and erythritol pathway.**

The results obtained in this study demonstrate that the metabolism is dependent on the carbon source available, and use of glucose and glycerol infers entry into the metabolism at different points. An overview of metabolism in the presence of glucose or glycerol is illustrated in Figure 5.

## Discussion

Batch cultivations of *Y. lipolytica* have been performed in this study to quantify cellular performance during growth on different concentrations of glycerol and a combination of glucose and glycerol as carbon sources. Focus has been on the interplay between substrate, oxygen availability and formation of products, and on quantifying physiological parameters allowing a detailed comparison of metabolism under the conditions employed.

### Growth and substrate utilisation

Table 1 summarises the data for the cellular performance indicators from all the cultivations performed. In batch cultures a maximum specific growth rate of around 0.3 h^-1^ was obtained on glycerol regardless of the concentration (10, 20 or 45 gL^-1^), whereas on glucose a growth rate of 0.24 h^-1^ was estimated.

This study shows detailed results from co-substrate submerged cultivation of *Y. lipolytica* in controlled bioreactors (Figure 4). A clear preference for glycerol over glucose was observed. This is further substantiated by the reduced growth rate on glucose compared to glycerol (Table 1). This preference of glycerol over glucose is converse to what has been previously observed with typically studied eukaryotic microbial cell factories (including *S. cerevisiae* and filamentous fungi such as *A. niger*), which exhibit carbon catabolite repression in the presence of glucose (Ruijter and Visser 1997Gancedo 1998). The observed results are likely to be a combination of carbon transport processes together with their overall regulation, illustrated in *S. cerevisiae*, by the hexose transporter family and the regulation of carbon utilisation through the MIG1 transcriptional regulator (Gancedo 1998, Ozcan and Johnson, 1999). It may be argued, therefore, that the glycerol preference by *Y. lipolytica* is a consequence of the genome only containing one hexose transporter but at least three genes coding for proteins associated with glycerol transport while lacking homologues for genes known to be involved in carbon catabolite repression (results from own BLAST search, data not shown).

Based on the data obtained in this study and previous knowledge of the metabolism of *Y. lipolytica* (Dulermo and Nicaud 2011) the steps from carbon source entry to product formation could be sketched (Figure 5). As illustrated in Figure 5, entry of glycerol to the central carbon metabolism can occur via 2 pathways both resulting in dihydroxyacetonephosphate (DHAP): (1) Via the conversion of glycerol to dihydroxyacetone succeeded a phosphorylation, or (2) Via a phosphorylation of glycerol into G3P, succeeded by an oxidation of G3P into DHAP. This oxidation can be carried out by two forms of glycerol-3-dehydrogenase, one bound to the mitochondrial membrane, and one freely dispersed in the cytosol. The enzyme located in the cytosol uses NAD/NADH as cofactors, and the membrane bound enzyme uses FAD/FADH_2_ as cofactors. From the dissolved oxygen plots (Figures 1, 2 and 3), it is clear that the utilisation of glycerol requires more oxygen than metabolisation of glucose alone. Our experimental data suggests that the second pathway, the G3P shuttle (see Figure 5), is the one primarily used by *Y. lipolytica*, due to the high oxygen requirement for biomass formation (Table 1) accompanying glycerol utilisation. In order to recycle the FAD/FADH_2_, *Y. lipolytica* must use ubiquinone as the acceptor, hereby transporting electrons across mitochondrial membrane and into the respiratory pathway (as outlined above). This could account for the high oxygen consumption, but without a similar high CO_2_ production when growing on glycerol compared to glucose.

### Polyol formation

Cultivations using various start concentrations of glycerol, demonstrated that polyol formation was initiated as a consequence of oxygen limitation (Figures 2 and 3). This correlates well with work on *Aspergillus niger*, where mannitol was shown to be produced during a combination of oxygen limitation and low specific growth rates (Diano et al. 2006;Pedersen et al. 2012). The reasoning for mannitol formation during oxygen limitation is that the excess NADH which cannot be oxidised through oxidative phosphorylation is used for mannitol biosynthesis to regain NAD^+^ (Diano et al. 2006). During growth on glycerol, this argument is further substantiated for *Y. lipolytica* as one additional mole of NADH is generated for each mole of glycerol metabolised, through activity of the G3P shuttle, resulting in additional NADH to be oxidised during growth on glycerol compared to glucose. This combined with the lower growth rate could explain the lack of polyol formation during growth on glucose.

*Y. lipolytica* has been shown to produce a variety of products including single cell oils, citric acid and sugar alcohols (Papanikolaou et al. 2002Levinson et al. 2007Beopoulos et al. 2009Rymowicz et al. 2009Rymowicz et al. 2010da Silva et al. 2012Fontanille et al. 2012Tomaszewska et al. 2012). Previous studies relate measurement of these product types to limitations of nutrient in the medium (particularly nitrogen or oxygen), but as the cultivations were operated at different scales and with different levels of process control and monitoring, it is difficult to fully understand the physical and chemical triggers leading to one product type or another. The specific isolate used in this study was capable of producing polyols (mannitol, arabitol and erythritol were measured). Only trace amounts of citric acid were produced and this may be due to the cultivation conditions employed, although the specific product spectra obtained in different studies may also be a result of strain variability in the various isolates applied (this has been illustrated in the study of Andre et al. 2009).

### Oxygen consumption and limitation

From Table 1 it appears that the oxygen requirement for biomass formation (Yxo) was independent of the use of carbon source (around 0.4 mol O_2_ cmol DW^-1^). It is intriguing that combining the two carbon sources resulted in a higher specific growth rate but a lower Yxo (reduced by 25%) than when single substrates were used. Oxygen limitation was not reached despite the high growth rate on glycerol and glucose combined. This indicated that co-metabolism of these substrates very efficiently generated biomass with regard to oxygen consumption. In addition, from the batch cultivations using 10 g L^-1^ glycerol (Additional file 2), oxygen limitation was very closely followed by c-limitation, showing that this amount of carbon source matches the oxygen availability under the set of conditions applied here. As indicated in Figure 1, metabolisation of glucose does not result in severe oxygen limitation. It may, therefore, be argued that through simultaneous utilisation of glucose and glycerol, *Y. lipolytica* is capable of maximising its metabolism with regards to oxygen availability promoting ATP generation by oxidative phosphorylation resulting in the observed improvement of growth rate without reaching oxygen limitation.

Considering the current necessity for biosustainable processes based on a variety of feedstock types, cell factories which can convert a diverse array of carbon sources are becoming increasingly attractive. The versatility of *Y. lipolytica* with regards to substrate utilisation makes it an attractive host for biorefinery applications and a relevant alternative to traditionally applied eukaryotic microbial cell factories. To improve and advance applications of *Y. lipolytica*, further detailed quantitative physiological assessment, such as has been provided by this study, is required. The development of genetic engineering tools and the application of omics approaches are rapidly advancing our understanding of this non-conventional yeast (Nicaud, 2012). The challenge now is facilitate cell factory improvement through omics driven approaches based on controlled submerged cultivations, in order to promote *Y. lipolytica* as a competitive yeast cell factory.

## Competing interests

The authors declare that they have no competing interests.

## Supplementary Material

Additional file 1 **Optical density as a function of time.** DO 100, DO 50 and DO 25 represents cultures carried out with 40 g L^-1^ glycerol and maintaining the DO at 100%, 50% and 25% respectively. In these cultures DO started at 100% and was allowed to fall to the set-point value, where after it was maintained for the remainder of the process. Oxygen supply was thus higher than in the standard glycerol cultures (run with 20 g L^-1^ or 40 g L^-1^ and 1vvm aeration throughout. This led to a prolonged exponential growth phase. For comparison, the optical density as a function of time for a cultures using 20 g L^-1^ glucose as carbon source is also shown. Click here for file

Additional file 2 Representative profile of glycerol concentration and dissolved oxygen (%) for the batch cultivation carried out with 10 g L^-1^ glycerol. Through linear regression (indicated on the graph), the volumetric glycerol uptake rate was estimated to be 0.019 ± 0.01. Click here for file
