# Supplementary figures and images for: Comparing cellular performance of Yarrowia lipolytica during growth on glucose and glycerol in submerged cultivations
Source: AMB Express. 2013 Oct 3;3:58. doi: 10.1186/2191-0855-3-58 (PMC3852309; doi:10.1186/2191-0855-3-58)

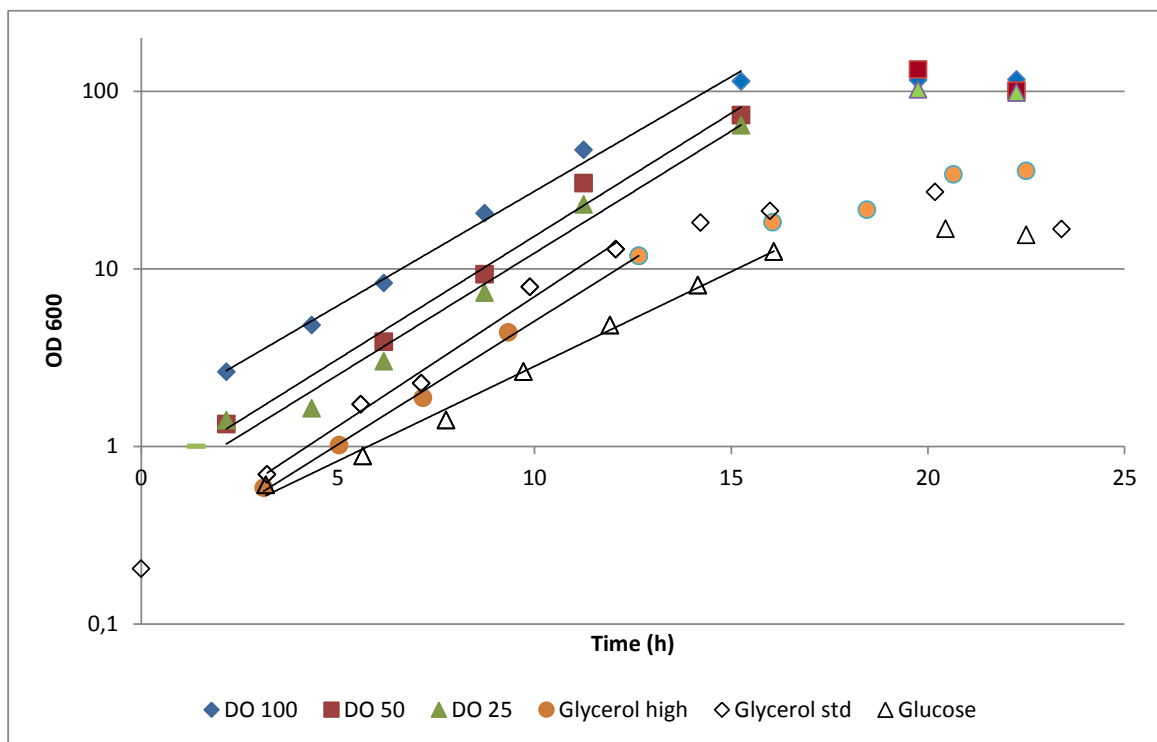

Supplement: Additional file 1 — Optical density as a function of time. DO 100, DO 50 and DO 25 represents cultures carried out with 40 g L-1 glycerol and maintaining the DO at 100%, 50% and 25% respectively. In these cultures DO started at 100% and was allowed to fall to the set-point value, where after it was maintained for the remainder of the process. Oxygen supply was thus higher than in the standard glycerol cultures (run with 20 g L-1 or 40 g L-1 and 1vvm aeration throughout. This led to a prolonged exponential growth phase. For comparison, the optical density as a function of time for a cultures using 20 g L-1 glucose as carbon source is also shown. [file 2191-0855-3-58-S1.pdf]

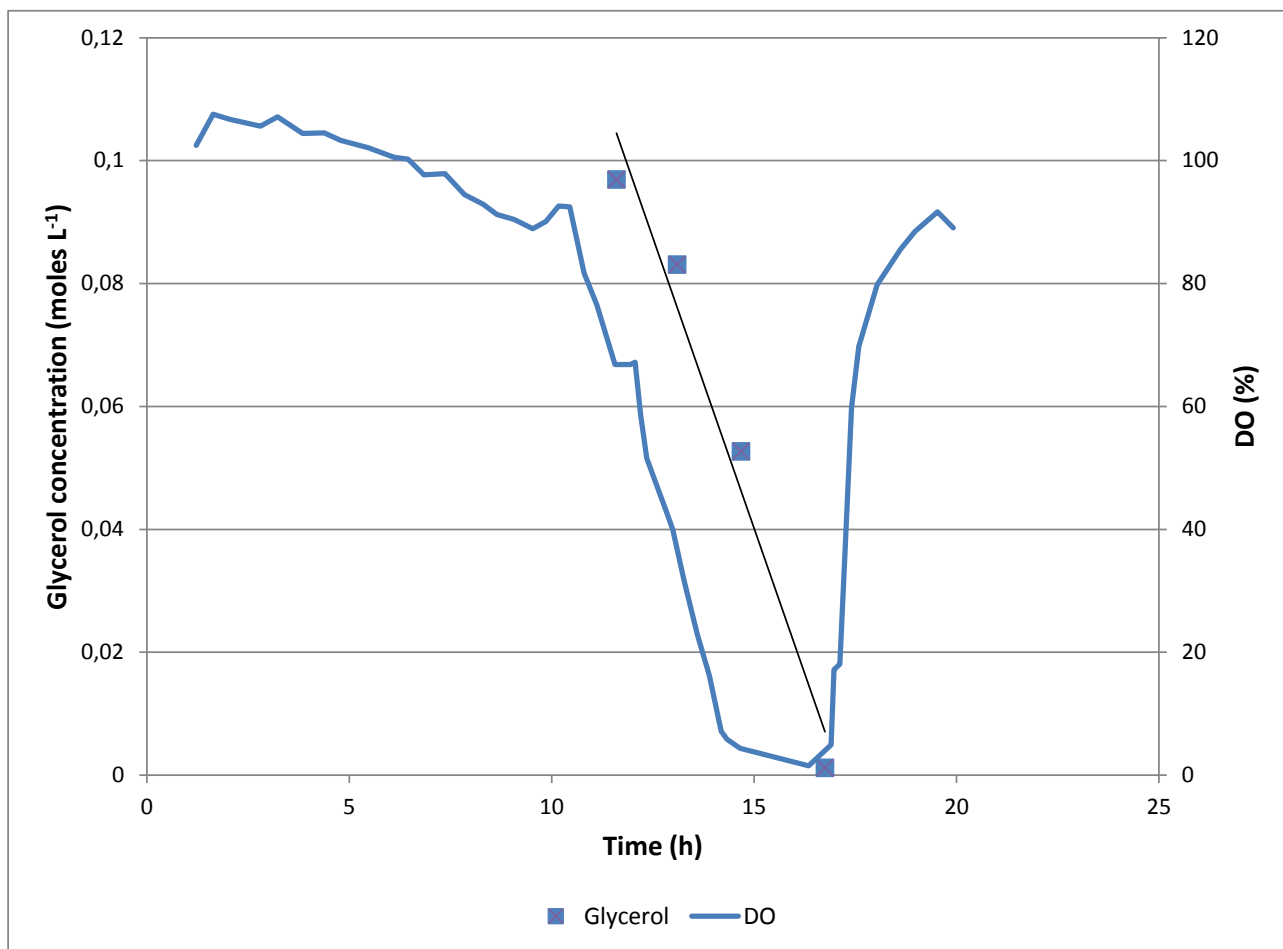

Supplement: Additional file 2 — Representative profile of glycerol concentration and dissolved oxygen (%) for the batch cultivation carried out with 10 g L-1 glycerol. Through linear regression (indicated on the graph), the volumetric glycerol uptake rate was estimated to be 0.019 ± 0.01. [file 2191-0855-3-58-S2.pdf]
